# Temporally consistent predominance and distribution of secondary malaria vectors in the *Anopheles* community of the upper Zambezi floodplain

**DOI:** 10.1038/s41598-021-04314-4

**Published:** 2022-01-07

**Authors:** Dónall Eoin Cross, Amy J. E. Healey, Niall J. McKeown, Christopher James Thomas, Nicolae Adrian Macarie, Vincent Siaziyu, Douglas Singini, Francis Liywalii, Jacob Sakala, Andrew Silumesii, Paul W. Shaw

**Affiliations:** 1 Institute of Biological, Environmental and Rural Sciences, Aberystwyth University, Aberystwyth, SY23 3FG UK; 2 Lincoln Centre for Water and Planetary Health, College of Science, University of Lincoln, Brayford Pool Campus, Lincoln, LN6 7TS UK; 3 Limulunga District Health Office, P.O. Box 910022, Mongu, Zambia; 4 School of Public Health and Health Systems, University of Waterloo, Waterloo, ON N2L 3G1 Canada; 5 Provincial Health Office, Western Province, P.O. Box 910022, Mongu, Zambia; 6 Ministry of Health, P.O. Box 30205, Lusaka, Zambia

**Keywords:** Biogeography, DNA sequencing, Entomology, Malaria

## Abstract

Regional optimisation of malaria vector control approaches requires detailed understanding both of the species composition of *Anopheles* mosquito communities, and how they vary over spatial and temporal scales. Knowledge of vector community dynamics is particularly important in settings where ecohydrological conditions fluctuate seasonally and inter-annually, such as the Barotse floodplain of the upper Zambezi river. DNA barcoding of anopheline larvae sampled in the 2019 wet season revealed the predominance of secondary vector species, with *An. coustani* comprising > 80% of sampled larvae and distributed ubiquitously across all ecological zones. Extensive larval sampling, plus a smaller survey of adult mosquitoes, identified geographic clusters of primary vectors, but represented only 2% of anopheline larvae. Comparisons with larval surveys in 2017/2018 and a contemporaneous independent 5-year dataset from adult trapping corroborated this paucity of primary vectors across years, and the consistent numerical dominance of *An. coustani* and other secondary vectors in both dry and wet seasons, despite substantial inter-annual variation in hydrological conditions. This marked temporal consistency of spatial distribution and anopheline community composition presents an opportunity to target predominant secondary vectors outdoors. Larval source management should be considered, alongside prevalent indoor-based approaches, amongst a diversification of vector control approaches to more effectively combat residual malaria transmission.

## Introduction

Malaria elimination strategies have been described as demonstrating a ‘*monolithic reliance*’ on indoor vector control, based on classical preferences of major *Anopheles* mosquito vectors for feeding on humans indoors at night^1^. While six major anopheline vector species are responsible for 95% of transmission across sub-Saharan Africa^2^ (‘primary’ vectors), such control strategies may be compromised by the presence of other feeding and resting behaviours^3^ such as propensities of minor (‘secondary’) vector species for outdoor and/or early biting. This may facilitate residual malaria transmission despite the decline of primary vector species^4,5^; in some contexts, species deemed ‘secondary’ vectors when considered at a continental scale can act locally as primary vectors and can sustain comparable levels of malaria transmission^4,6^. Indoor-focused approaches such as long-lasting insecticide nets (LLINs) and indoor residual spraying (IRS) have been found to not only increase insecticide resistance^7,8^ but also to drive within-species shifts in behaviour^9^, plus shifts in vector community composition^10–13^ towards taxa whose behaviours enable them to elude such interventions. As well as responding to interventions, anopheline communities are subject to the influence of environmental conditions over various spatial and temporal scales. Potential future shifts in temperature^14^ and hydrological regimes^15^ may profoundly influence the distribution of malaria vectors and consequent transmission dynamics^16,17^. The twin threats posed by residual transmission and potential climate change effects on mosquito vector communities have been highlighted^14,18^ as major challenges to future malaria control, and emphasise the importance of entomological surveillance^19^.

Entomological surveillance is vital to monitor vector community responses to interventions and climate change, and to characterise the species assemblage to ensure that interventions are locally relevant^20^. The reliable identification of vector species is increasingly achieved through genetic barcoding alongside traditional morphology^21^, permitting the association of bionomic traits with individual species^22–24^ which enables an increasing diversity of interventions to be tailored to specific vector behaviours^25^. Genetic barcoding is also a powerful means for species identification of larval stages, which are frequently morphologically indistinguishable, with direct application to larval source management (LSM). Diverse LSM approaches^26–31^ have been successfully implemented over small scales of time and space, but operational deployment at large scale is challenging^32^. To optimise the timing and location of LSM implementation, it is vital to understand seasonal variations in mosquito community composition and distribution at the appropriate temporal and spatial scale^33–35^.

Cross et al*.*^36^ employed DNA barcoding of anopheline larvae sampled in an extensive spatial survey to reveal an unexpectedly high prevalence of secondary vector species in a region of persistent residual malaria transmission in western Zambia, with partitioning of species across ecological zones and stability of patterns observed over dry and wet seasons. The present study aims to build on that work by combining geographically extensive surveys of larval habitats and DNA barcoding of larvae sampled in the same region in the following year to assess inter-annual consistency of anopheline vector community composition, ecological zonation and seasonal patterns. Furthermore, barcode data for adults collected both by de novo sampling in this study, and previously by Orba et al*.* (2018^37^ and 2021, pers. comm.) and Wastika et al*.*^38^ over a contemporaneous 5-year period in the same region of western Zambia are assessed for linking of adult and larval patterns. Finally, as the sampled period spanned a wet season in 2019 that was unusually early and dry, and followed by the most severe drought for at least 20 years^39^, we also discuss the results within a climate change context given the increased frequency and severity of droughts predicted for the region by climate change models.

## Results

### Larval habitat sampling

**Figure 1:**
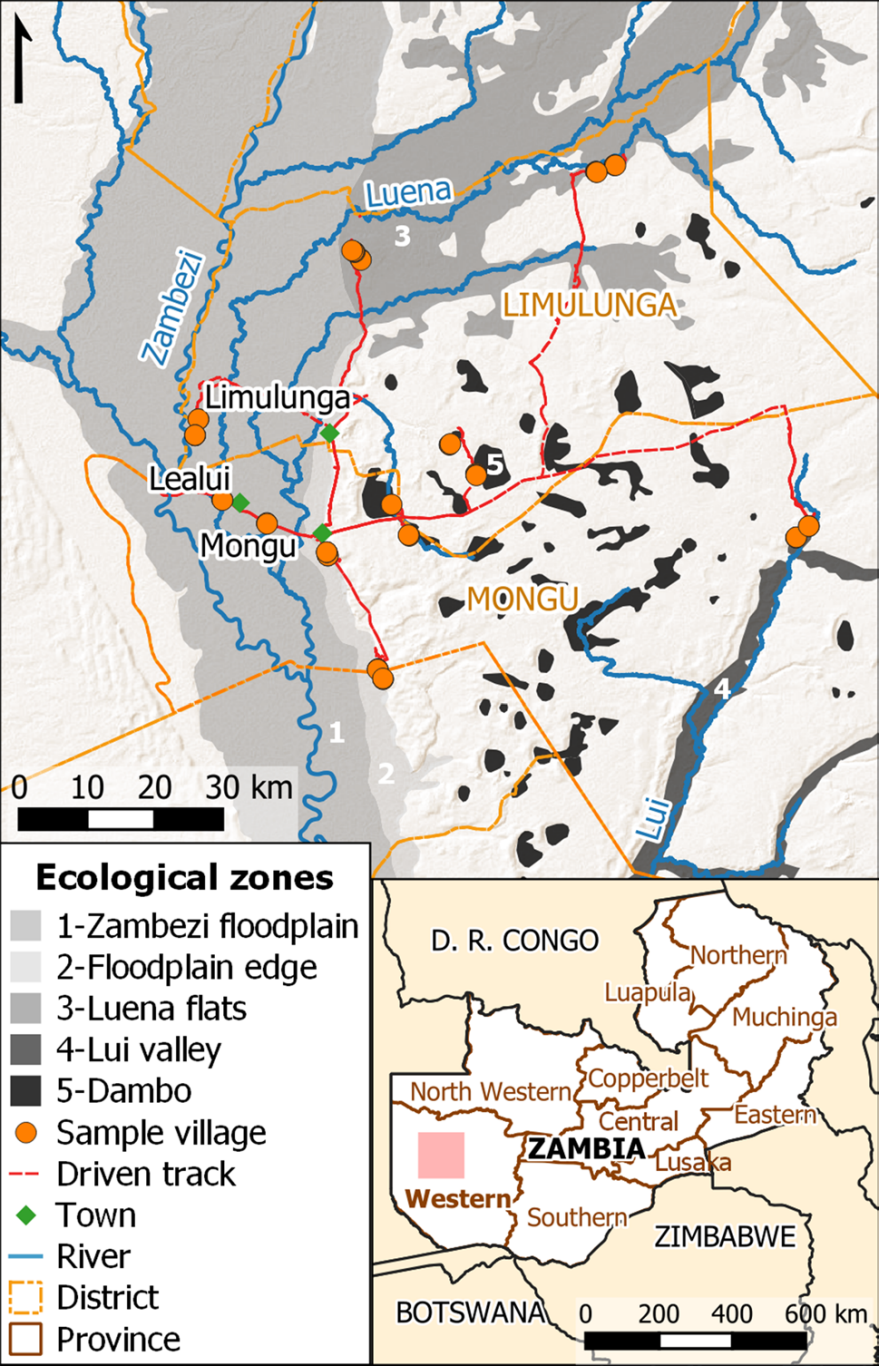
Ecological zones and entomological survey locations in Western Province, Zambia. 1 = Zambezi floodplain; 2 = Floodplain edge; 3 = Luena flats; 4 = Lui valley; 5 = dambos. Larval surveillance and adult mosquito trapping undertaken after the peak of the 2019 wet season. Pink rectangle in inset indicates study map extent. Map drawn in QGIS^94^ (v. 3.18.3-Zürich; https://www.qgis.org); basemap: ESRI Shaded Relief (2021).

Sampling was undertaken in May–June 2019 after the peak of the wet season, comprising 32 transects totalling over 11 km (Fig. 1). Transects ranged from 68 to 647 m in length (mean ± standard deviation (SD): 348 ± 146 m); water was encountered in a similar proportion of transect points in 2019 as in the previous wet season (79.5% and 80.1%, respectively), and the average number of dips taken per wet transect point in 2019 was comparable to 2018 (22 ± 8.2 and 23 ± 5.4, respectively).

*Anopheles* larvae were encountered across a diverse range of larval habitats, from flooded grasslands to seepage zones, and permanent water bodies in dambos to open, transient pools on sandy substrates. Water bodies were predominantly vegetated (see Fig. 3 in Hardy et al*.*^40^), with common vegetation types including grasses (*e.g. Echinochloa* spp*.* and *Oryza* spp*.*) and papyrus (*Cyperus* spp.); landscape and vegetation have been characterised in detail elsewhere^40–43^. *Anopheles* occurred in 74.1% of wet transect points in 2019, which was significantly lower than the previous wet season (85%; Odds Ratio (OR) 0.505, 95% CI 0.330–0.771, *p* < 0.001). An average of 1.31 (± 3.8) mosquito larvae were encountered per dip, of which approximately a quarter were anophelines (mean 0.32 ± 0.91 per dip). There was no significant difference between total anophelines per transect point between 2018 and 2019 wet seasons (median of 2 per 10 dips for both years; independent samples median (ISM) test 0.95, df 1, *p* = 0.33), although the proportion of larvae classified as late stage was significantly higher in 2019 than 2018 (36% and 23%, respectively; OR 1.9379, 95% CI 1.043–3.602, *p* = 0.036).

**Figure 2:**
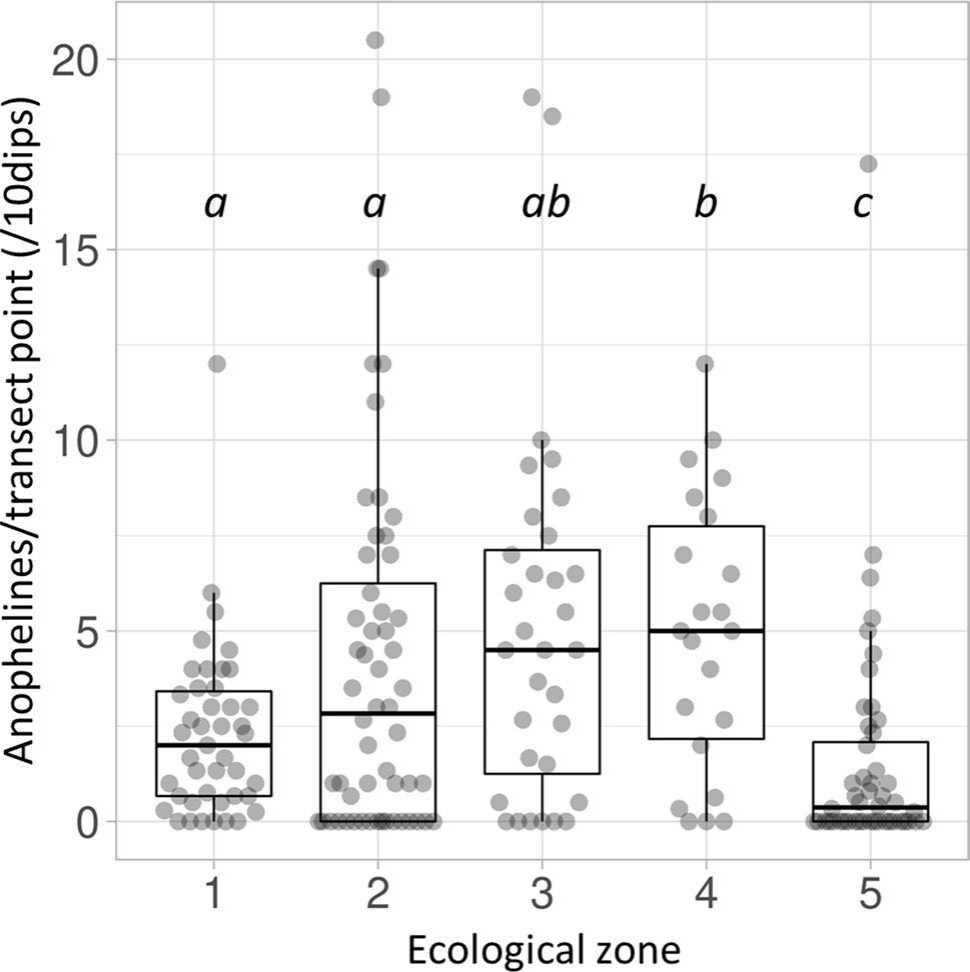
Anopheline larval abundance within 5 defined ecological zones (see Fig. 1 and Methods) in western Zambia. 1 = Zambezi floodplain; 2 = Floodplain edge; 3 = Luena flats; 4 = Lui valley; 5 = dambos. Total value per transect point (standardised per 10 dips) represented by dots; boxplot indicates interquartile range and median (horizontal line), with whiskers extending to minimum and maximum values within 1.5 times IQR. Transect points *n* = 220; anophelines n = 1557. Median values for ecological zones that do not share a letter are significantly different (independent-samples median test with stepwise step-down comparisons; adjusted p < 0.001).

Ecological zone comparisons are made between total abundance values per transect point, standardised per 10 dips due to variation in water body size necessitating significant differences between ecological zones in total dips per transect point (ISM test 31.401, *df* 4, *p* < 0.001). Anopheline larvae were encountered in all ecological zones across the study area, with up to 20.5 per transect point (standardised per 10 dips). The median standardised total anophelines per transect point differed significantly between ecological zones (ISM test 21.536, *df* 4, *p* < 0.001), with fewer anophelines in dambos than in any other zone, and more in Lui valley transect points than in the Zambezi floodplain (stepwise step-down (SSD) post-hoc analysis; adjusted (adj) for multiple comparisons *p* < 0.05; Fig. 2).

### Genetic identification: larvae

**Figure 3:**
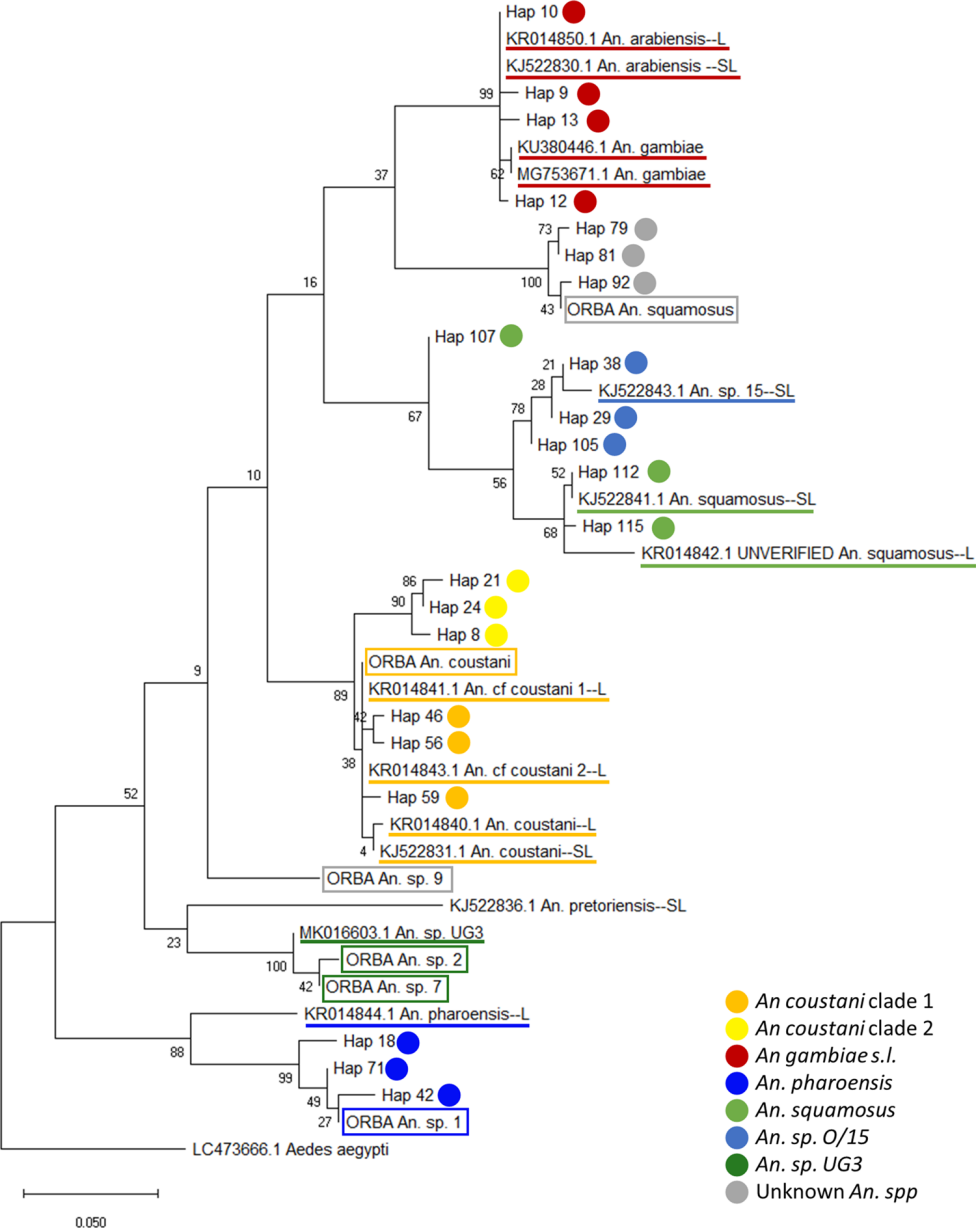
Maximum Likelihood phylogenetic tree of representative cytochrome *c* oxidase I (COI) sequences from *Anopheles* sampled in western Zambia. Consensus tree constructed in MEGA X from haplotypes of a 300 bp fragment of the COI region of mitochondrial DNA, using 100 bootstrap replicates. Non-singleton haplotypes preferred, where available; up to three haplotypes selected at random per taxon. Species identities assigned from > 95% BLAST similarity to COI and/or ITS2 sequences published on GenBank are indicated with coloured dots and prefixed ‘Hap’. Sequences from adult mosquitoes caught in the region (Orba et al., 2021, pers. comm.) are prefixed ‘ORBA’; species name indicates morphologically-derived identity, while coloured box indicates molecularly-derived identity. Published reference sequences labelled with GenBank accession number and species name; suffix denotes source paper. ‘–C’ denotes Ciubotariu et al.^44^; ‘–L’ denotes Lobo et al.^22^; ‘–SL’ denotes St Laurent et al.^23^. Species also indicated with coloured underline. Tree drawn to scale; branch lengths measured in number of substitutions per site. Full tree available in Supplementary Information as Fig. S1.

Over 50% of field-surveyed anopheline larvae were collected for genetic analysis (*n* = 855/1557), and a viable DNA sequence from COI and/or ITS2 regions was obtained from 658 specimens (Table 1; the remainder did not amplify using COI or ITS2 primers, potentially due to sub-optimal preservation of DNA in the field). More than two-thirds of these specimens (*n* = 443) were identified to species based on both COI and ITS2 sequences with ≥ 95% similarity to GenBank accessions. An additional 20% of specimens (*n* = 129) matched with ≥ 95% identity with GenBank COI sequences, but their corresponding ITS2 sequences returned no match at ≥ 95% similarity with a GenBank sequence. Each remaining specimen (*n* = 86) was assigned a species identity based on an above-threshold match of either its COI or ITS2 sequence to GenBank accessions, with the exception of 18 specimens (2.7% of the total) whose closest matches were to *Anopheles* sequences but at similarities below the threshold; these 18 were subsequently designated ‘unknown *Anopheles* species’. Maximum likelihood (ML) tree analysis of the 119 resolved COI haplotypes corroborated the species identities assigned from matches to GenBank sequences by demonstrating statistically well-supported clusters nested with reference specimen sequences of known morphological identity (Fig. 3; Supplementary Fig. S1). *An. gambiae* complex species clustered together but were not resolved into species from COI phylogeny.

**Table 1:** Species identities assigned to *Anopheles* larvae based on mitochondrial and nuclear DNA sequences.

Species/taxon	COI-assigned	ITS2-assigned	Consensus ID	%
*An. coustani*	489	376	527	80.1
(of which *An. coustani* clade 2)	(0)	(0)	(145)	(22.0)
*An. pharoensis*	72	54	72	10.9
*An. gambiae s.l*	13	14	14	2.1
(of which *An. arabiensis*)	(0)	(7)	(7)	(1.1)
(of which *An. gambiae* s.s.)	(0)	(7)	(7)	(1.1)
*An. squamosus*	11	11	13	2.0
*An. species* O/15^20^	10	8	11	1.7
*An. species* UG3	0	3	3	0.5
Unknown *An.* species	15	154	18	2.7
Total	610	620	658	100

Identities inferred from above-threshold matches to cytochrome *c* oxidase I (COI) and/or internal transcribed spacer region 2 (ITS2) sequences on GenBank (National Center for Biotechnology Information nucleotide database). Larval sampling undertaken May–June 2019 in Limulunga and Mongu districts, Zambia.

**Figure 4:**
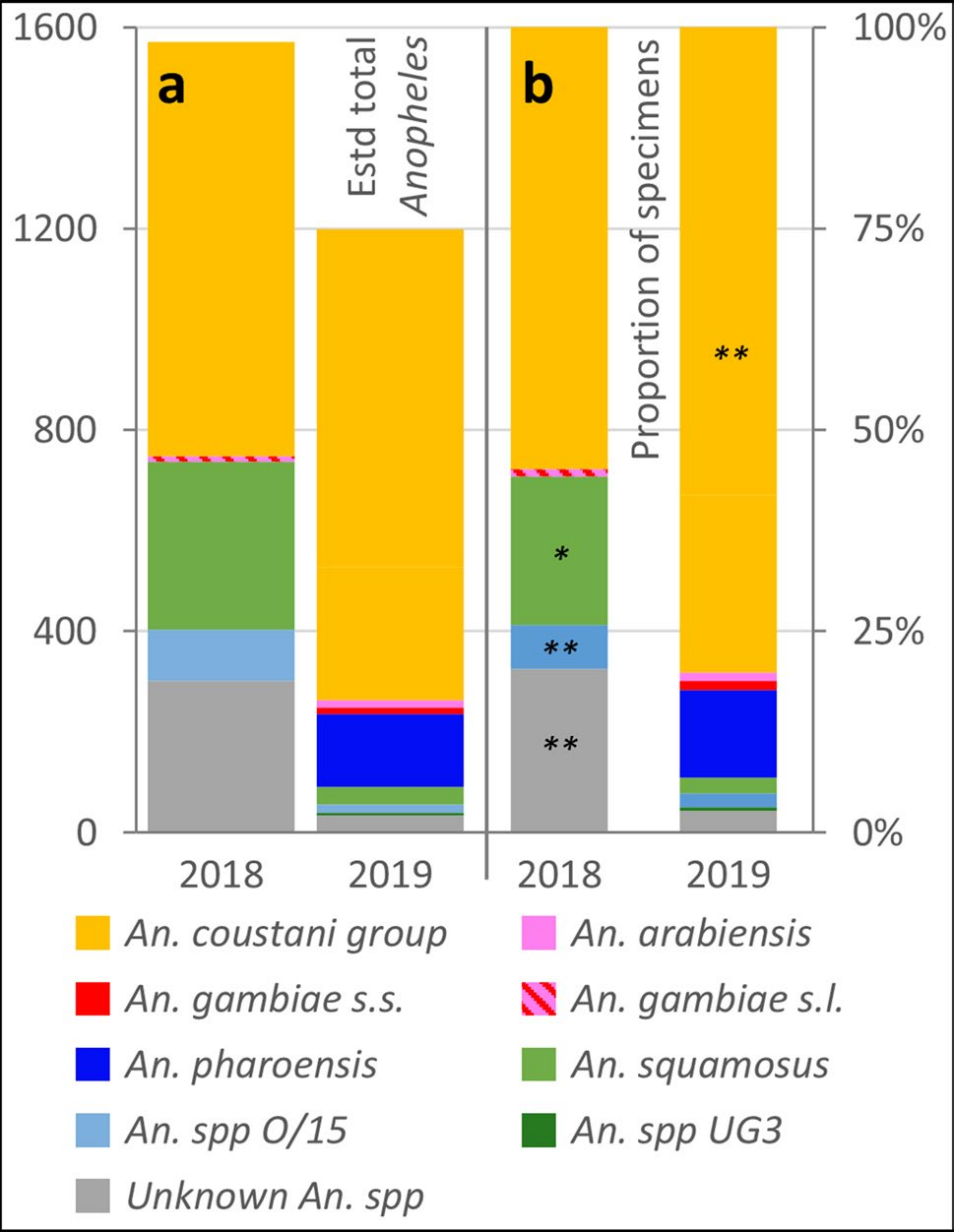
Abundance and species composition of larval anopheline community in western Zambia in two consecutive wet seasons (2018 and 2019). (**a**) Estimated total abundance calculated by applying species proportions from DNA sequence-identified subset of sampled larvae (see **b**) at each transect point to the total surveyed larvae per transect point, and summing for all transect points. (**b**) Proportion of specimens assigned species identities from matches to COI and/or ITS2 DNA barcode sequences from GenBank (2018: *n* = 748; 2019: *n* = 658). Significant differences between years in proportion of of specimens represented by each individual taxon indicated by asterisk(s); *p < 0.01; **p < 0.001 (*z*-test for independent proportions). Groups represented by hatched bars were not resolved to individual member species in 2018. ‘Unknown *An.* spp’ indicates that match to *Anopheles* reference sequences fell below 95% similarity threshold.

Within the sample of 658 sequenced larvae, a large majority were identified as *An. coustani* (80.1%; Table 1; Fig. 4). Two distinct genetic clades could be further separated within *An. coustani*; the first consisted of specimens with both COI and ITS2 sequences matching with ≥ 95% identity to accessions for the species, whilst the second clade (comprising 22% of the total sample) consisted of specimens with above-threshold matches to COI reference sequences but below-threshold matches to any GenBank ITS2 sequence. This second *An. coustani* clade also forms a well-supported cluster in the ML tree (Fig. 3; for full ML tree see Supplementary Fig. S1). *An. pharoensis* was the next most abundant species at 10.9% (Fig. 4), followed by small numbers of *An. gambiae* sensu lato (2.1%), *An. squamosus* (2.0%), *An. species* O/15^23^ (1.7%), and 3 specimens (0.5%) designated as *An. species* UG3, signifying ‘unknown group 3’ based on similarity to designations made in southern and central Africa by Ciubotariu et al*.*^44^. *An. gambiae* sensu lato could be further resolved into *An. arabiensis* (1.1%) and *An. gambiae* sensu stricto (1.1%, hereafter *An. gambiae*) based on ITS2 sequences.

### Geographic distribution of larvae

**Figure 5:**
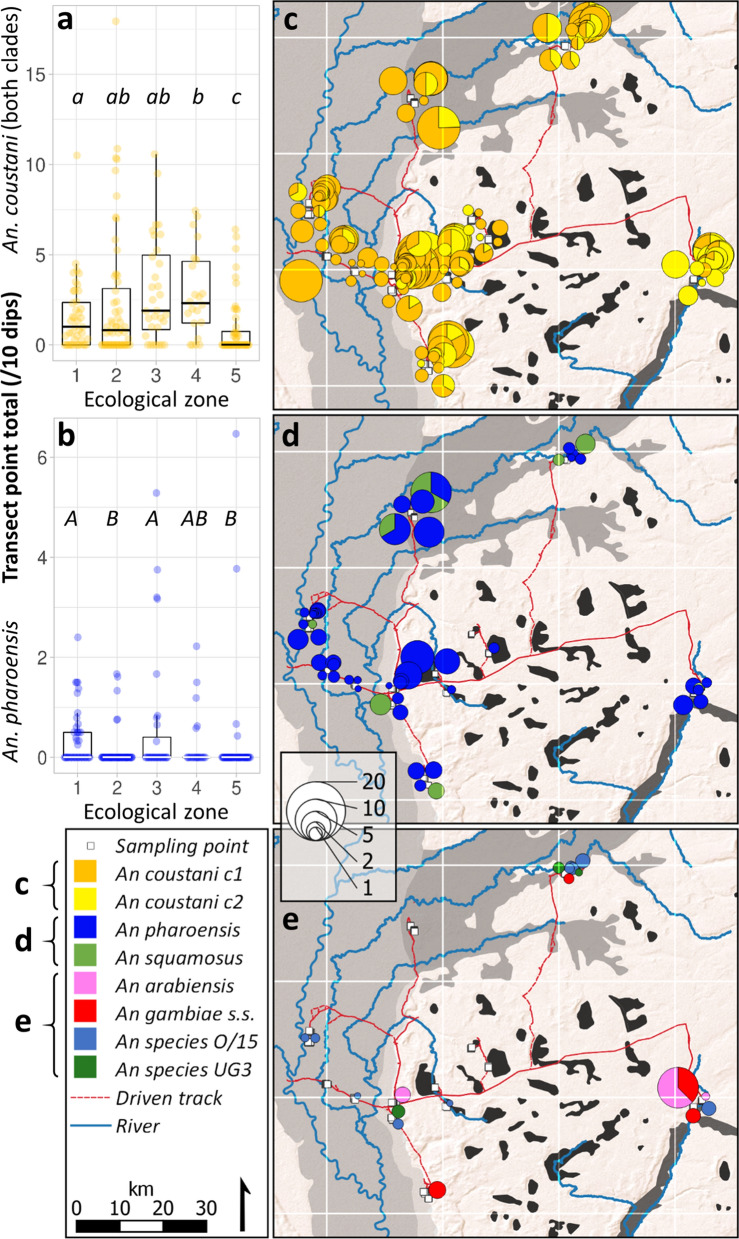
Distribution of anopheline larvae across ecological zones after peak of 2019 wet season. Species identities assigned from matches to COI and/or ITS2 sequences from GenBank. (**a–b**) Species abundance across ecological zones. Ecological zones as per Fig. 1 and boxplot format as per Fig. 2. Ecological zones not sharing a letter are significantly different; lower case letters = independent samples median test; upper case letters = Kruskal–Wallis distribution test; stepwise step-down comparisons with adj *p* < 0.001. **(c-e**) Spatial distribution of species across ecological zones (see Fig. 1). Symbol area proportional to estimated total larvae of mapped species per transect point (product of total anopheline count and taxa proportions in subsample), standardized per ten dips. Map drawn in QGIS^94^ (v. 3.18.3-Zürich; https://www.qgis.org); basemap: ESRI Shaded Relief (2020).

**Figure 6:**
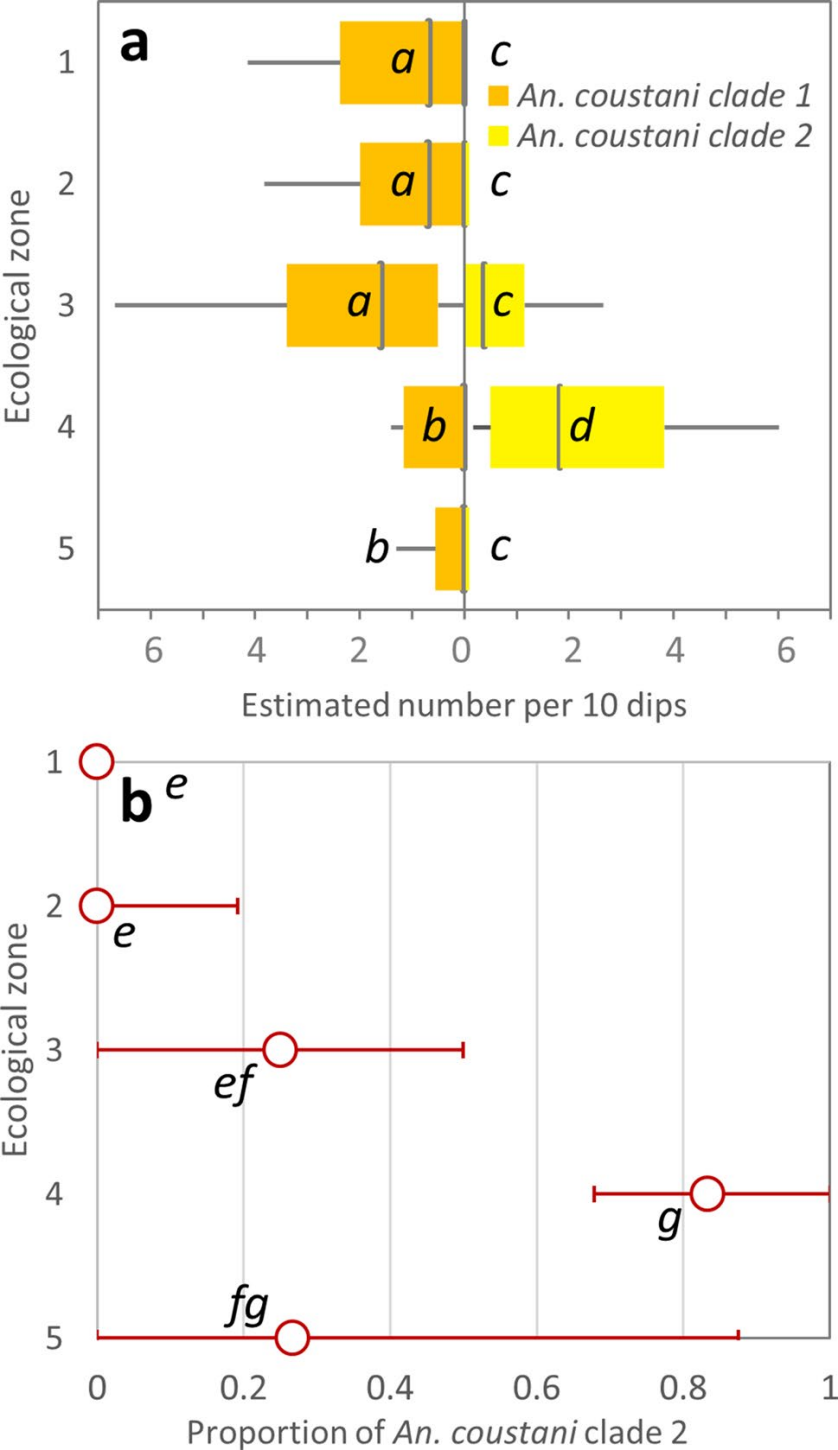
Composition of *An. coustani* clade 1 and clade 2 larval populations in western Zambia, 2019. (**a**) Abundance of clade across ecological zones. Boxplot represents interquartile range and median (vertical line) estimated total of each clade per 10 dips; whiskers extend to minimum and maximum values within 1.5 × IQR. Ecological zones as per Fig. 1. (**b)** Median proportion of clade 2 in *An. coustani* specimens. Calculated for all transect points where at least one clade present (*n* = 128); median proportion indicated by circles, using secondary axis; whiskers indicate IQR. Ecological zones not sharing a letter within two separate comparisons in (**a**) (LHS and RHS) and in (**b**) are significantly different; independent samples median test; stepwise step-down comparisons with adj p < 0.001.

*An. coustani* was distributed ubiquitously across ecological zones (Fig. 5c). A significantly lower median per transect point was found in dambos than in the Zambezi and Luena floodplains and the Lui valley, and a significantly higher median in the Lui valley compared to the Zambezi floodplain and its edge (Fig. 5a; ISM test 26.150, *df* 4, *p* < 0.001; SSD adj *p* < 0.05). Within *An. coustani*, however, clade 1 exhibited a significantly lower median in the Lui valley (and dambos; median = 0) than all other habitats (Fig. 6; 0.6–1.592; ISM test 20.036, *df* 4, *p* < 0.001; SSD adj *p* < 0.05). Conversely, the clade 2 median was significantly higher in the Lui valley than all other ecological zones (Fig. 6; median 1.822 compared to 0–0.369; ISM test 35.518, *df* 4, *p* < 0.001; SSD adj *p* < 0.05). The median proportion of estimated *An. coustani* populations composed of clade 2 individuals was significantly higher in the Lui valley than all ecological zones except dambos, and the proportion in dambo populations exceeded those of Zambezi floodplain and floodplain edge populations (Fig. 6; ISM test 34.057, *df* 4, *p* < 0.001; SSD adj *p* < 0.05).

*An. pharoensis* was also encountered in every ecological zone (Fig. 5d), albeit in fewer transect points (21%) than *An. coustani* (65%). Its distribution in Zambezi and Luena floodplains was significantly different from dambo and floodplain edge habitats (Fig. 5b; Kruskal–Wallis *H* = 21.392, *p* < 0.001; mean rank former > latter, SSD adj *p* < 0.05). The primary vector complex *An. gambiae* s.l. was found in floodplain edge (*n* = 2 *An. gambiae*), Luena flats (*n* = 1 *An. gambiae*) and Lui valley ecological zones (*n* = 6 *An. arabiensis* and *n* = 4 *An. gambiae*; Fig. 5e). *An. squamosus* was found in all ecological zones except for dambos (Fig. 5d), and small numbers of An. species O/15 were present in every ecological zone, occurring in 5% of wet transect points; there was no significant difference in distributions between ecological zones for either of these species. Three specimens of *An. species* UG3 were found, from samples in floodplain edge and Luena flats ecological zones (Fig. 5e). The peri-urban anopheline larval community sampled along Kambule stream (*n* = 19 transect points; 55 anophelines) exhibited similar composition to the overall sample community, with *An. coustani* accounting for 76% of the estimated species total, *An. pharoensis* 18%, and *An. arabiensis* and unknown *An. species* making up the remainder (4% and 2%, respectively).

### Adult mosquito sample

Paired BG-Malaria traps were deployed at a total of 63 households across 18 villages over 9 trap nights. Traps ran overnight for *c*. 18 h (mean duration 17h41 ± 1h00; mean start 16:29 ± 0h42; end 10:11 ± 0h40). One hundred and twenty-six trap nights yielded 58 anophelines which were predominantly female (93%), with a considerable majority trapped outside (71%), including all males (*n* = 3; excluded from subsequent analyses). The distribution of catches amongst the traps was highly clustered; 32 traps contained one or more anophelines, and a single outside trap accounted for 25% of the total catch. Although modest in extent and compromised by the unusually dry wet season, we report and interpret these results as they coincided geographically and temporally with larval surveys.

Fifty-four adult female specimens were identified to 6 species from above-threshold matches to COI and/or ITS2 DNA sequences (Table 2), confirmed by high bootstrap support of clustering within phylogenetic ML tree analysis (Supplementary Fig. S1). *An. species* UG3 dominated the adult sample (46%), with the majority found outdoors, although a single outdoor trap accounted for half of this outdoor total. Over half of *An. species* UG3 specimens were identified by matches of both COI and ITS2 sequences to accessions linked to Jones^45^ and Ciubotariu et al*.*^44^, respectively, whilst the remaining specimens were identified from matches to either these ITS2 (24%) or COI (20%) sequences. ITS2 sequences permitted differentiation between species within the *An. funestus* group that could not be resolved based solely on COI sequences. The typically anthropophilic primary malaria vector species *An. funestus* (24% of sample) was predominantly trapped indoors (77% of specimens). *An. gambiae* adults were found exclusively indoors, whilst *An. coustani*, *An. arabiensis* and *An. rivulorum* (*An. funestus* group member) were found exclusively outdoors. One specimen was assigned ‘unknown *An. species*’. None of the adults were bloodfed or reported positive amplifications from PCR aimed at detecting *P. falciparum* sporozoites.

**Table 2:** Composition of sample of adult female Anopheles from western Zambia after 2019 wet season peak.

Species	Indoor	Outdoor	Total
*An. coustani*	0	7	7
*An. arabiensis*	0	2	2
*An. gambiae*	4	0	4
*An. funestus*	10	3	13
*An. rivulorum*	0	1*	1
*An. species* UG3	3	22	25
Unknown *An. species*	0	2	2
Total	17	37	54

Species identities based on above-threshold matches to COI and/or ITS2 DNA sequences on GenBank. ‘Unknown *An. species*’ denotes specimen whose sequence matched to *Anopheles* reference sequences, below 95% similarity threshold. *Sex unknown as morphological characters missing from damaged specimen.

### Geographic distribution of adult mosquitoes

**Figure 7:**
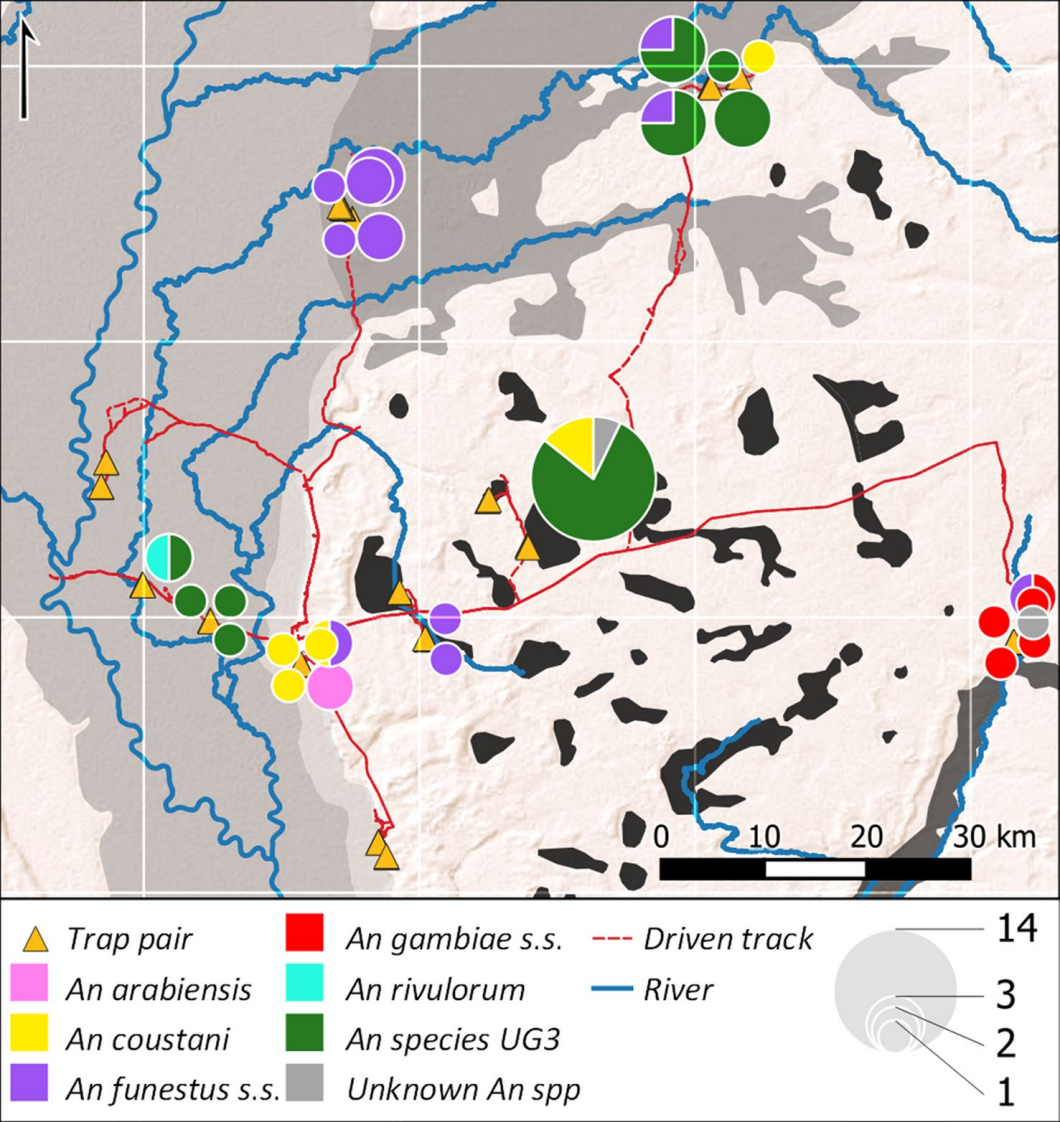
Distribution of adult *Anopheles* sampled using BGM traps in western Zambia. Specimens (*n* = 54) identified from above-threshold matches of COI and/or ITS2 sequences to GenBank accessions. Pie chart area proportional to total catch per trap pair (indoor + outdoor). Ecological zones as per Fig. 1. Map drawn in QGIS^94^ (v. 3.18.3-Zürich; https://www.qgis.org); basemap: ESRI Shaded Relief (2020).

Adult anopheline catches exhibited a high degree of within-village similarity, whilst varying substantially between villages (Fig. 7). Although *An. species* UG3 numerically accounted for almost half of the adults sampled, this was a consequence of an unusually high catch in one dambo village; it was found in one-third of villages where *Anopheles* adults were caught (*n* = 4 of 12). Samples from two villages in the Luena flats and one in a dambo were exclusively *An. funestus*, while the species was also found on the floodplain edge and the Lui valley. *An. coustani* was found in 4 villages, whilst *An. gambiae* adults were confined to 2 villages in the Lui valley; *An. arabiensis* and *An. rivulorum* occurred solely in one floodplain edge and one Zambezi floodplain village, respectively (Fig. 7).

### Inter-annual comparisons: 2017–2019

**Figure 8:**
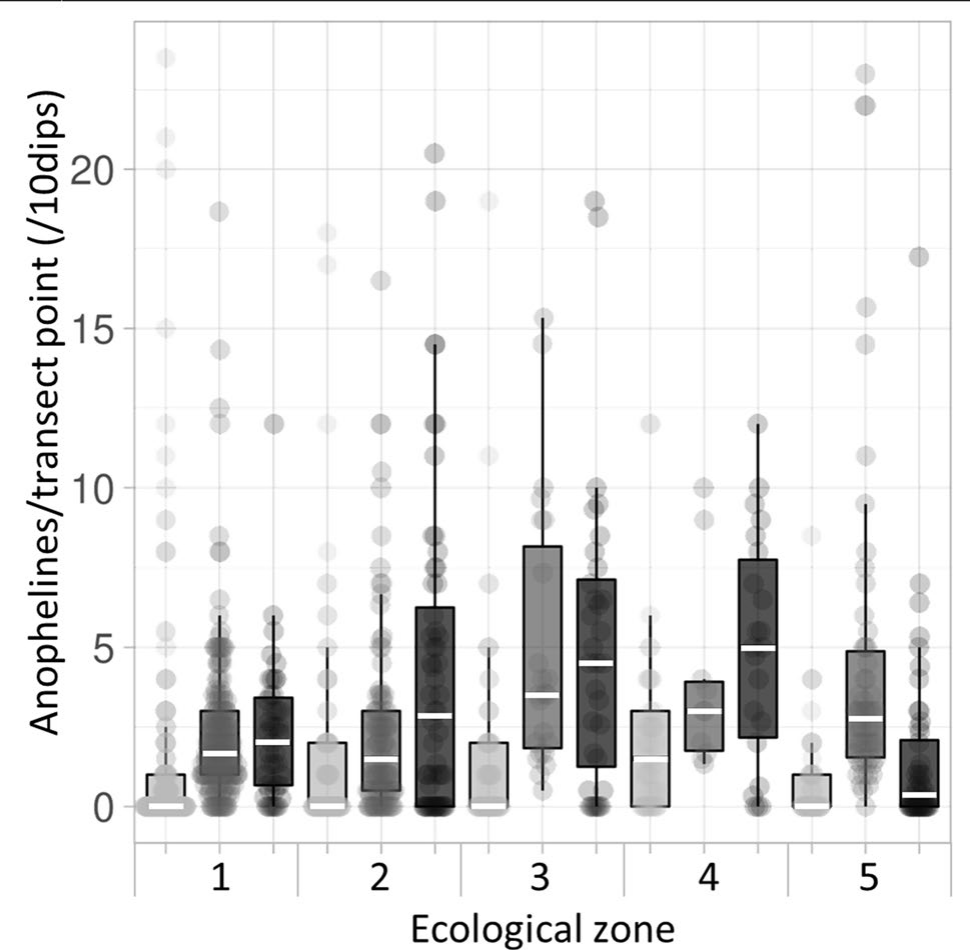
Distribution of *Anopheles* larvae across ecological zones in western Zambia. Sampling undertaken in the dry season of 2017 (light grey) and after peak of the wet season in 2018 (mid grey) and 2019 (dark grey). Total anopheline larvae per transect point (standardised per ten dips) represented by dots; boxplot indicates interquartile range and median (white horizontal line), with whiskers extending to minimum and maximum values within 1.5 times IQR. 2017: *n* = 407 transect points; 2018: *n* = 340; 2019: *n* = 220. Ecological zones as per Fig. 1.

Despite the considerable difference in the magnitude and timing of the wet season between these years, the relative abundance of all anophelines in each ecological zone in 2019 was more consistent with the 2018 wet season than the dry season of 2017 (Fig. 8). *An. coustani* comprised a larger proportion of the sample in the 2019 wet season than in 2018, whilst *An. pharoensis* and *An. squamosus* combined, *An.* species O/15*,* and unknown *An.* species occupied a larger proportion in 2018 than in 2019 (Pearson χ^2^ 150.325; df 5; *p* < 0.001 and post-hoc *z*-tests for independent proportions; *p* < 0.001; Fig. 4b). Comparisons between anopheline species distributions in the 2017 dry season (larvae^36^), after the peak of the 2018 wet season (larvae^36^), and after the peak of the 2019 wet season (adults and larvae) revealed considerable inter-annual consistency in the spatial distribution of some anopheline species where sampling effort coincided. For example, *An. coustani* adults were caught in 6 outdoor traps in 2019 (Fig. 7), and larvae of this species were found in the nearest wet transect point to each of these traps (Fig. 5c). The presence of *An. coustani* on 2019 transects was always preceded by the presence of *An. coustani* group larvae on the corresponding transect in the previous 2018 wet season, and usually also in the dry season in 2017 (see Fig. 5c and Cross et al*.*^36^). Four larval transect points contained *An. gambiae* in 2019 (Fig. 5e); half of these occurred within 100 m of 3 of the 4 households where adult *An. gambiae* were caught indoors in the Lui valley (Fig. 7). *An*. *arabiensis* larvae were also found in the same Lui valley location in 2019 (Fig. 5e), within 250 m of three transect points which contained *An. gambiae* s.l. larvae in 2017 (2017 sample identities were resolved to species complex level only, as ITS2 sequences were not then available for all specimens^36^). In the floodplain edge ecological zone, adult *An. arabiensis* were trapped outdoors within 250 m of a transect point where *An. gambiae* s.l. larvae were found in 2018^36^. Two *An. gambiae* larvae were encountered in floodplain edge habitat in 2019 analogous to two floodplain edge locations where *An. gambiae* s.l. larvae occurred in the same season in 2018^36^. Similarly, although *An. species* O/15 larvae were comparatively fewer in 2019, they occurred in Zambezi floodplain habitats comparable to those surveyed by adjacent transects in 2018; several 2019 occurrences (Fig. 5e) were within 600 m of where the species was encountered in the preceding wet season^36^. In the Luena flats, locations positive for *An. species* O/15 larvae on overlapping 2018 and 2019 transects occurred within 100 m of each other (Fig. 5e and Cross et al*.*^36^).

### Adult data derived from previous studies

Trapping of adult mosquitoes in Mongu district between 2014 and 2017 yielded 1829 anophelines mainly outdoors^37,38^, and additional trapping in 2018–19 provided an additional 596 anophelines (Orba et al*.,* 2021, pers. comm.). The majority of anophelines in each of six sampling periods from 2014 to 2019 were identified as *An. coustani*, ranging from 49 to 100% of the sample, whilst 13% of the specimens across all periods were *An. squamosus* (Fig. 8). *An. gambiae* s.l. mosquitoes were the sole primary vector species detected, occurring only in 2017 when they constituted < 0.5% of the sample. Although anopheline species count data from these studies^37,38^ were not published, the data collected during the studies and subsequently (Orba et al., 2021; pers. comm.) are provided in the Supplementary Information to this paper (Supplementary Table 1), along with six COI gene sequences (Supplementary Dataset S1).

**Figure 9:**
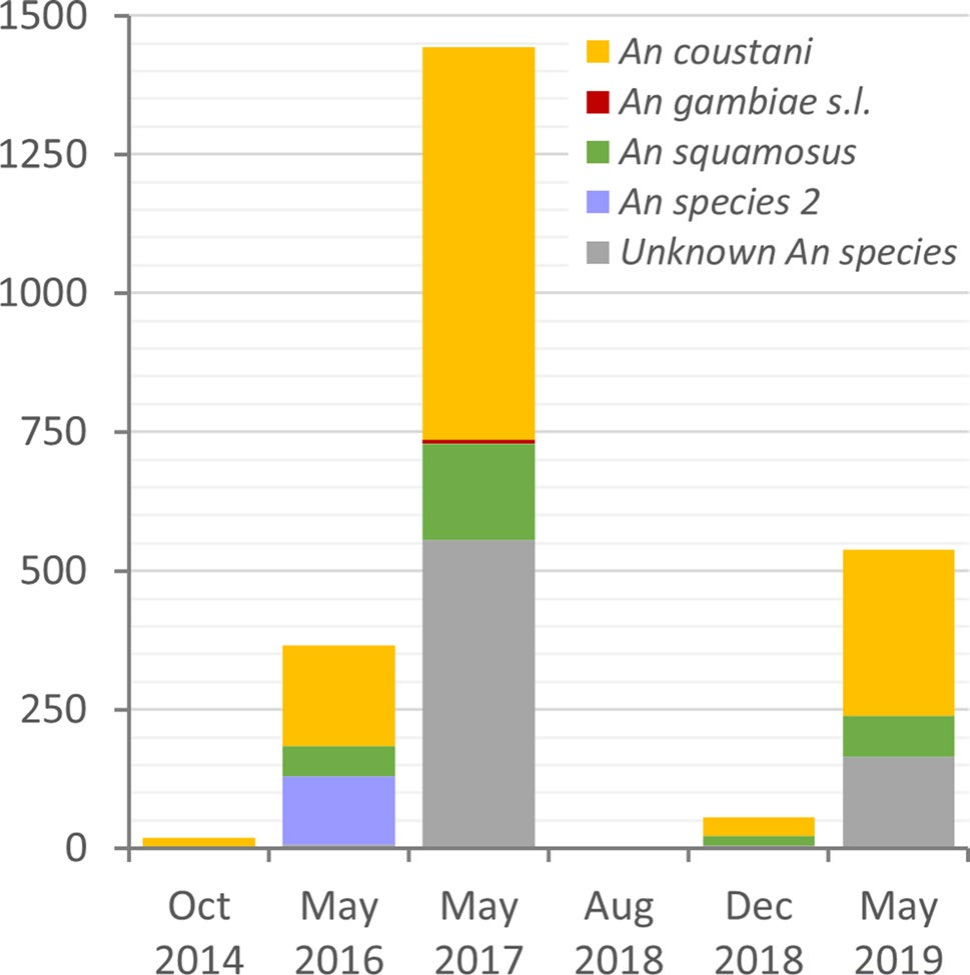
Species composition of adult anophelines trapped in multiple locations in Mongu district, Zambia. Samples collected mainly outdoors using CDC and BG-Sentinel traps, and identified morphologically, with a subset of identities confirmed by sequencing of COI. Data sources: Orba et al.^34^, Wastika et al.^35^, Orba et al. (2021, pers. comm).

There was strong support for the morphological identification of adult *An. coustani* as the DNA sequences obtained from reference samples within the independent dataset (Orba et al*.*, 2021, pers. comm.) clustered with sequences on GenBank and *An. coustani* sequences from the present study (Fig. 3; Supplementary Fig. S1). The time series of data indicates the consistent dominance of *An. coustani* in the region in both dry and wet seasons (Fig. 9).

## Discussion

Geographically extensive larval mosquito sampling was undertaken across multiple ecological zones in western Zambia over three years, including one dry and two wet seasons, and data were integrated with adult mosquito catches from this study and an independent series of collections over a longer timeframe^37,38^ (Orba et al*.,* 2021, pers. comm.). This combined dataset revealed a marked temporal consistency in the composition of the *Anopheles* mosquito community across western Zambia, and also consistency in geographic distribution of species across larval habitats in the region. This spatial consistency is evident both at landscape scale, across large ecological zones in a study area over 3500 km^2^, and also at fine scale whereby larvae were frequently encountered within 10–100 s of metres of congruent records from a preceding study in the region^36^. The community consistency was even more notable given the significant variation in ecohydrological conditions between the 2018 wet season and the 2019 season surveyed in the present study. Barotseland experienced an anomalously early and unusually dry wet season in 2019, such that widespread drought in the ensuing dry season was the most intense and severe for at least 20 years^39^, whilst Zambezi River discharge in 2018 was the second highest recorded for almost 30 years^36^. Extensive surveys of *Anopheles* larvae across diverse habitats in this study, coupled with objective molecular identification to species revealed a paucity of primary vector species, with support for this finding provided by an independent dataset of adult mosquitoes caught in the study area in the same time period^37,38^ (Orba et al*.*, 2021, pers. comm.; see Supplementary Table S1 & Dataset S1).

*An. coustani* was found to be by far the most ubiquitous and abundant species across the region, constituting an estimated 78% of all surveyed anopheline larvae (Fig. 4), compared with 52% of surveyed anophelines in the preceding 2018 wet season and 65% in the dry season of 2017 which comprised *An. coustani* group larvae^36^. Although *An. coustani* is often rare in entomological surveys^24^, in recent studies it has been found to dominate local anopheline communities at locations in the western Kenyan highlands^46^ and Madagascar^47^, and was the second most prevalent species at locations in Ethiopia^48^ and rural southeast Zambia^49^. The species was distributed ubiquitously across our study area, but with higher abundance in the Luena flats corresponding to a significantly higher median in this ecozone in the previous wet season^36^. The lower abundance of *An. coustani* in dambo habitats in this study is likely to reflect the comparatively lower encounter rate of all anophelines in this ecological zone in 2019, whilst in 2018 anopheline abundance in dambos was comparable to that of habitats in the Luena flats and Lui valley^36^. Whilst *An. coustani* is relatively understudied^22,44^, typical larval habitat preferences have been broadly characterised as permanent water bodies with clear water and aquatic/semi-aquatic vegetation^50,51^; it has been found in vegetated aquatic habitats in association with long grass^52^ which are a feature of the survey landscape in western Zambia, particularly in waterlogged areas of the Luena flats which retain water year-round^40^. Adult *An. coustani* group mosquitoes maintain a year-round presence in Kenya^53^ and Ethiopia, although they reached peak density in the drier months following the rainy season in the latter^48,54^; *An. coustani* abundance may have been higher in the current study than in surveys following the 2018 wet season^36^ due to the drier conditions experienced in western Zambia in 2019^39^. Although the long-term data from adult trapping in the centre of our study area (Fig. 9) have a limited seasonal resolution, they indicate the persistent presence of the *An. coustani*; whilst abundance fluctuated between years, *An. coustani* was consistently predominant, providing strong corroboration for the findings from larval surveillance.

The *An. coustani* group includes multiple species, including *An. coustani, An. crypticus, An. tenebrosus* and *An. ziemanni* ^51,55,56^; some are morphologically indistinguishable in some life stages, and the use of molecular methods are advocated particularly to differentiate the cryptic *An. crypticus* from *An. coustani* ^57^. While COI and ITS2 sequences have been published for the other species, none are available for *An. crypticus*. In the current study corresponding ITS2 sequences were obtained from 99% of specimens identified as *An. coustani* based on their COI sequences. This ITS2 dataset revealed two distinct genetic clades within *An. coustani* in western Zambia: one whose ITS2 sequences matched GenBank *An. coustani* sequences with > 95% similarity, and another with ITS2 similarity to GenBank accessions of < 95%. There was a high level of spatial overlap between clades, but significant differences in the proportion of the two *An. coustani* clades between ecological zones raises the possibility that the clades occupy different ecological niches within the study area. Ciubotariu and colleagues^44^ also report this phenomenon amongst morphologically-identified adults, reporting a clade whose closest match for known *An. coustani* ITS2 sequences was ~ 80%. Members of both clades in that study were also reported to overlap spatially in northern Zambia and neighbouring D.R. Congo, and contained specimens positive for human blood meals and *P. falciparum* confirming their roles as secondary malaria vectors^44^. It is possible that *An. coustani* clade 2 in this study represents *An. crypticus,* although we note that our sequences do not match the ITS2 sequences of two *‘coustani*-like’ species reported by Lobo et al*.*^22^ which could also represent the species.

The presence of *An. pharoensis* in 2019 (reported here) despite its absence in 2018^36^ is most likely attributable to a difference in genetic resolution between studies rather than a demographic/biological signal, as species assignment in the previous study was based almost entirely on COI which confers less discriminatory power between *An. pharoensis* and *An. squamosus* than ITS2^21,24,58^. The more extensive ITS2 sequencing performed here therefore permitted identification of *An. pharoensis*, and was strongly supported by phylogeny (Fig. 3; Supplementary Fig. S1). This artefact does not affect our main findings, as pooling of *An. squamosus* with *An. pharoensis* in 2019 did not alter the ecological zone partitioning exhibited by *An. pharoensis* alone. Significantly higher *An. pharoensis* abundance in Zambezi and Luena floodplain ecological zones in 2019 corresponded with significantly higher prevalence of *An. squamosus* (potentially including *An pharoensis* individuals) in these habitats and along the floodplain edge in 2018^36^.

Eight of the nine anopheline species identified in this study have previously been implicated as malaria vectors, whilst the vector status of *An. species* UG3 and of our *An. coustani* clade 2 is unknown. In Kenya at least half of 17 species in one study tested positive for *P. falciparum* or were known vectors^23^, while 12/21 species in a more recent study were potential vectors^24^, and 12/18 in eastern Zambia^22^ tested positive for *P.* *falciparum.* The number of adult anophelines sampled in the present study was severely limited by the unseasonally dry conditions, and the small sample did not contain bloodfed mosquitoes. Nonetheless, our adult samples were captured in traps baited with CO_2_ and lures emulating human odours, so it can be assumed that they were seeking bloodmeals. *An. coustani* has tested positive for *P. falciparum* in multiple settings^4,22,46,47,59–62^, and marked tendencies for exophagy^46,53,54,59^ (as evident in this study) and early biting^48,54^ may render it largely unaffected by indoor-focused interventions. Parasite-positive *An. pharoensis* is implicated in maintaining dry season malaria transmission in Ethiopia^54^ and this species also favours outdoor biting^54,59^, as does *An. squamosus*^63^ which was found to have a *Plasmodium* positivity rate equal to that of primary vectors on Madagascar^47^ and an unexpected degree of anthropophily in southern Zambia^64^. Recently *An. species* O/15 was shown for the first time to harbour malaria parasites following outdoor trapping in the western Kenyan highlands^24^. *An. rivulorum* is considered to be a less efficient vector than its sibling species *An. funestus*, but nonetheless carries the malaria parasite^22,65^, whilst *An. arabiensis, An. gambiae* and *An. funestus* are classical primary vector species^56^, and the latter two were caught predominantly indoors in the present study.

Comparatively few studies have examined the inter-annual dynamics of anopheline communities across the landscape scales at which larval source management would need to be implemented^66^, given the dispersal capacity of *Anopheles* vector species^67^. Studies frequently seek to quantify the effect of seasonality on vector communities^48,53,54,68,69^, examine a limited number of locations at high temporal resolution^9,70^ or investigate community change in response to an explicit intervention^10,71^. Spatially explicit descriptions of community composition and distribution at relatively large scales of space and time are scarce; previous studies have largely focused on adult mosquitoes and variously reported temporal shifts^9^ or consistency^70,72,73^. The current study reports a high degree of consistency in anopheline community composition and distribution over three consecutive years. *An. coustani* larvae are shown to be widely distributed and to predominate in the community in both dry and wet seasons, while the traditional primary vector species *An. arabiensis* and *An. gambiae* comprise a consistently low proportion of the community (< 3% of larvae) and manifest a distinct small scale geographic clustering which is consistent across years. Due to the limited numbers (*n* = 54), the adult sample is unlikely to be representative of the anopheline community, particularly given the absence of samples from 30% of larval sampling locations and the skewing of the sample by a single anomalous catch constituting 25% of sampled adults. Nonetheless, the adult sample corroborates the *An. gambiae* complex hotspot in the Lui valley. Despite the high prevalence of *An. species* UG3 in the adult sample, it represented < 0.5% of the larval sample. The adult sample also contained two species unrepresented in 2019 larval samples (*An. funestus* and *An. rivulorum*), perhaps due to the preference of the former species for heavily vegetated water bodies^74^ and tolerance of submergence^75^ reducing its representation in dipped larval samples^76^. However, an independent time series of extensive catches of adult mosquitoes from the centre of the study area (*n* > 2400; ^37,38^ and Orba et al*.*, 2021, pers. comm.) provides substantial corroboration for the anopheline community composition described from the larval surveillance presented here and previously^36^. This dataset confirms the consistent dominance of *An. coustani* and the notable absence of significant numbers of primary vector species in both dry and wet seasons.

The anopheline community across Barotseland, western Zambia, experiences dramatic seasonal fluctuations in environmental conditions in a highly dynamic ecosystem driven by seasonal flooding and rainfall regimes^40^. Although anophelines were encountered in a lower proportion of water bodies after the 2019 wet season than after the preceding 2018 wet season, the larval community was composed of a higher proportion of late stage larvae, suggesting increased productivity in the drying down phase of the accelerated hydrological year in 2019. This has been reported in other settings, potentially as abundant habitats become smaller and warmer: larval abundance was higher in drying streams than other habitats in Kenya and Tanzania^52^, increased with falling river levels in Sudan^77^, and both larval abundance and adult productivity increased in the early dry season in Kenya^78^. Many of the species encountered in the present study area exhibit preferences for relatively permanent water bodies and community composition was consistent during three survey periods incorporating seasonal and inter-annual disparities in ecohydrological conditions. Nonetheless, nuanced intra-annual variations due to species-specific responses to seasonal changes and the changing importance of different water body types over the hydrological year may occur over finer temporal scales^68^. As climate change is predicted to increase the frequency of meteorological extremes in Zambia^79^, it is important to monitor the response of the *Anopheles* community to extreme conditions beyond the typical spectrum of seasonal variations. Analysis of historical data has revealed a downward trend in Zambezi river discharge since the 1950s^80^, and Zambia is projected to experience reduced rainfall and increased temperature in the twenty-first century^81^; a recent drought in southern Zambia reduced the abundance of *An. arabiensis* by an order of magnitude^69^. A significant and extreme drought in western Zambia followed the larval surveys presented in this study, and expanded entomological surveillance is advocated to characterise the effects of such events on the assemblage of anopheline species in this ecosystem.

In an area of persistent malaria transmission despite long-term indoor vector control efforts, extensive larval sampling of the anopheline community over consecutive years has revealed the numerical and spatial dominance of species widely accepted to be secondary vectors of malaria and a marked paucity of primary vectors. This finding underlines the importance of diversifying vector control approaches to counteract species whose behaviours may permit them to evade widespread use of indoor-centric interventions. The consistent dominance of these secondary vector species was strongly supported by independent surveillance of adult mosquitoes in the area over 5 years, and geographic distributions of candidate vector species were robust to seasonal and inter-annual variations in ecohydrological conditions. This temporal consistency of larval community structure in western Zambia suggests that this may be a setting where larval source management strategies could be highly effective, compared to other regions where the larval community fluctuates substantially over time.

## Methods

### Study area

The study area in Limulunga and Mongu districts in Western Province, Zambia has been characterised hydrologically^40^ and five main ecological zones (Fig. 1) described in detail^36^. Briefly, it is dominated by the Barotse or upper **Zambezi floodplain** (ecological zone 1) which is a heterogeneous grassland-wetland mosaic which receives overbank flow from the Zambezi River and its tributaries^82,83^. The **floodplain edge** (zone 2) along the base of the eastern escarpment contains persistently wet seepage zones^43^ fed by water from higher ground. Flooding from a highly branched tributary of the Zambezi River persists in wet grassland in the **Luena flats**^43^ (zone 3), whilst another tributary provisions distant seepage wetland^42^ habitats in the more defined **Lui valley** (zone 4) which dissects higher ground east of the Barotse floodplain. Shallow, frequently waterlogged depressions known as **dambos** (zone 5) form an important persistent agricultural and hydrological resource in the region in their own right^84^, as well as sustaining fertile regions along the floodplain edge below the scarp.

### Mosquito sampling: larvae

Field surveys of anopheline larvae were undertaken in water bodies in the five ecological zones after the peak of the wet season in May–June 2019, following the sampling strategy employed in Cross et al*.* (2021)^36^. Two health facilities were selected in each ecological zone (except in the remote Lui valley, where *n* = 1) and selection of two sample villages adjacent to each facility was guided by local knowledge of current hydrological conditions. Previous sample locations of Cross et al*.*^36^ were prioritised for re-sampling where hydrological conditions permitted (almost 80% of 2019 transects), but some were unsuitable for entomological sampling in 2019 due to the earlier and lower peak of the wet season and were replaced by adjacent villages. Radial line transects were sited from each village and sampling points were located at 100 m intervals along each transect. Water bodies encountered within a 5 m radius of these pre-defined points or within 5 m of the transect line at intermediate locations were geolocated with a GPS handset (Garmin eTrex) and surveyed for mosquito larvae. Up to 40 dips per transect point were taken using standard 350 ml dippers (Bioquip, USA) by employing a purposive dipping strategy^27,30,85^ to search for larvae within suitable microhabitats. Dip contents were examined in a white plastic tray after a settling period, and counted after morphological differentiation into anophelines and culicines. Up to 12 *Anopheles* larvae per sampling point were collected and stored individually in 95% ethanol for genetic analyses.

In addition to sampling of ecological zones described above and previously^36^, larval surveys were undertaken along Kambule stream within Mongu town to characterise the anopheline community in this peri-urban setting.

### Mosquito sampling: adults

Simultaneous indoor and outdoor sampling of adult mosquitoes was undertaken during the larval sampling period using an adaptation of the BG-Sentinel trap (Biogents, Germany^86^) optimised for anopheline mosquitoes and known as the BG-Malaria trap^87^. Each trap was suspended from a tripod and inverted with its opening 40 cm above ground level, baited with a cartridge of BG-Lure synthetic attractant (Biogents, Germany) and with CO_2_ produced by fermentation of yeast (40 g) and brown sugar (500 g) in 2 l of water and released within the trap. One indoor-outdoor pair of traps was deployed at each sample household to survey both endophagic and exophagic mosquitoes; the indoor trap was located close to the foot of an occupied bed and the outdoor trap positioned in the lee of the house or nearby vegetation, adjacent to outside sitting areas and away from sources of smoke or disturbance. Trap houses were preferentially located on the outer fringes of the village due to the ‘edge effect’ identified in some studies^88–90^, downwind of the village centre as female anophelines are postulated to fly upwind in search of human hosts^91^.

Traps were deployed in late afternoon and ran until the battery and fermentation mixture were disconnected the subsequent morning. Each trap catch bag was closed and labelled, and mosquitoes killed by freezing. Each catch was subsequently examined under a dissecting microscope and screened morphologically by genus, sex and bloodfeeding status. All anophelines were retained and stored individually in 95% ethanol.

### Adult mosquito data derived from previous studies

Adult mosquitoes were sampled in several districts across Zambia between 2012 and 2017 by Orba et al*.*^37^ and Wastika et al*.*^38^. Outdoor trapping was undertaken between 2014 and 2017 in Mongu district, where additional trapping was also undertaken in 2018–19 (Orba et al*.*, 2021; pers. comm.). Trapping was undertaken at several locations adjacent to Mongu town and Namushakende, on the floodplain edge *c.* 20 km south of Mongu, for an average of 5 consecutive nights per season using CDC and BG-Sentinel traps; see Orba et al*.*^37^ for details. Specimens were identified to species morphologically, with mtDNA COI gene sequences obtained from a subset of individuals for confirmation of identity (Orba et al*.*, 2021; pers. comm.).

### DNA barcoding species identification

Genetic analysis of larval and adult samples by mtDNA COI and nuclear ITS2 sequencing followed Cross et al.^36^. The species identity of each specimen was inferred from BLASTn search results yielding COI and/or ITS2 sequence similarity of ≥ 95% to sequences in GenBank (National Center for Biotechnology Information). Adoption of this standard sequence similarity threshold was vital to ensure comparability with previous results^36^ and those from other studies^22,23^. Species identities were further assessed by position of clustering within a maximum likelihood inferred phylogeny of the COI dataset and published confirmed species identity reference sequences^22,23,44^, constructed in MEGA v.10.0.5^92^, with statistical support calculated using 100 bootstrap replicates. Additionally, adult female anophelines were screened for the presence of *P. falciparum* sporozoites by PCR amplification using primers for the protozoan^93^.

### Data handling and statistical analyses

Field and molecular data were compiled in a database (MS Access) and linked to geographic locations in QGIS v. 3.18.3-Zürich^94^. Exploratory analyses were undertaken by plotting data points from individual dips together with aggregated summary measures (median values per year/ecological zone) using PlotsOfData^95^. The proportion of the genetically-identified subsample of anopheline larvae composed of each species individually was applied to the total anopheline count at each transect point to obtain the estimated total of each species at each transect point. Statistical analyses were undertaken in SPSS^96^; comparisons of field statistics (*e.g.* total anopheline larvae per transect point) and sequence-derived species data (*e.g.* estimated *An. arabiensis* per transect point) were made between consecutive wet seasons (2018^36^ and 2019) using Odds Ratios^97^ and Pearson’s Chi-squared test. Sampling in 2017^36^ is referenced as the dry season comparator. Within-year comparisons between ecological zones were made using non-parametric statistics: independent samples median (ISM) test and Kruskal–Wallis *H*, both with stepwise step-down (SSD) post-hoc comparisons and adjusted (adj) *p* value for multiple comparisons.

### Ethical approval

An ethical approval waiver was provided by the University of Zambia’s Biomedical Research Ethics Committee (Ref 018–08-17) as the research did not involve human subjects. The Barotseland Royal Establishment granted their approval for entomological surveys to be conducted in and around villages in the study area. District Health Office staff accompanied the field survey team; at the beginning of each day’s fieldwork, the survey team checked in with the nearest health facility and sought permission from village chiefs to undertake fieldwork following introductory discussions. After full explanation of the adult trapping procedure in an appropriate language (usually siLozi), written informed consent was obtained from householders who volunteered to participate, and LLINs issued where absent from indoor trapping houses. All methods were performed in accordance with relevant guidelines and regulations.

## Supplementary Information

Supplementary Information 1.

Supplementary Information 2.

Supplementary Information 3.

## Data Availability

The COI and ITS2 sequence datasets generated by and analysed in the current study are available in the NCBI GenBank nucleotide archive with accession numbers OL619678-OL619792 and OL583776-OL583807 for larval and adult COI sequences, respectively and OL621257-OL621744 and OL621788-OL621838 for larval and adult ITS2 sequences. A full maximum likelihood phylogenetic tree of COI haplotypes from this study and from Orba et al*.* (2021, pers. comm.) is provided in Supplementary Information (Fig. S1). *Anopheles* count data and six COI sequences from the latter source are provided in Supplementary Table S1 and Supplementary Dataset S1.
