# Supplementary material for: Temporally consistent predominance and distribution of secondary malaria vectors in the Anopheles community of the upper Zambezi floodplain
Source: Sci Rep. 2022 Jan 7;12:240. doi: 10.1038/s41598-021-04314-4 (PMC8742069; doi:10.1038/s41598-021-04314-4)
Supplement: Supplementary file 1 — Supplementary Information 1. [file 41598_2021_4314_MOESM1_ESM.pdf]

Cross, D.E., Healey, A.J.E., McKeown, N.J., Thomas, C.J., Macarie, N.A., Siaziyu, V., Singini, D., Liywalii, F., Sakala, J., Silumesii, A. & Shaw, P.W.

Supplementary Information: Adult mosquito data derived from previous studies

Supplementary Dataset S1

COI sequences from adult *Anopheles* mosquitoes trapped in Mongu District, Western Province, Zambia by Orba *et al.* (2018)[1], Wastika *et al.* (2020)[2] and Orba *et al.* (2021, pers. comm.).

Methods detailed in *Suppl_Methods_&_Table_S1.pdf.*

>COI-1

AACTTTATATTTTATCTTTGGAGCTTGAGCCGGAATAGTGGGAACTTCTTTAAGAATTCTAATTCGAGCTGAAT
TAGGTCACCCTGGAGCTTTTATTGGTGATGATCAAATTTATAATGTAATTGTAACTGCTCATGCTTTTATTATAA
TTTTTTTCATAGTAATACCTATTATAATTGGAGGGTTTGGAAATTGATTAGTTCCTTTAATATTAGGAGCCCCTG
ATATAGCTTTCCCTCGAATAAATAACATAGGATTTTGAATACTTCCGCCTTCATTAACTTTACTAATTTCTAGTA
GTATAGTACAAAATGGGGCAGGAACAGGATGAACTGTTTACCCTCCCCTGTCTTCGGGTATTGCTCATGCCGG
AGCTTCTGTTGATTTAGCTATTTTTTCTTTACATTTAGCAGGTATTTCATCTATTTTAGGAGCTGTAAATTTTATT
ACTACAGTGATTAATATACGATCCCCTGGTATTACATTAGACCGAATACCATTATTTGTATGATCAGTTGTTATT
ACAGCTGTATTATTACTTCTATCTTTGCCGGTTCTAGCTGGAGCAATTACTATATTATTAACAGATCGAAATTTA
AATACATCCTTCTTTGACCCAGCAGGAGGAGGAGATCCAATTTTATACCAA

>COI-2

AACATTATACTTTATTTTCGGTGCTTGAGCTGGAATAGTAGGAACTTCACTAAGAATTCTTATTCGAGCTGAAT
TAGGTCATCCCGGAGCATTTATTGGAGATGATCAAATTTATAATGTAATTGTTACTGCTCATGCTTTTATTATAA
TTTTCTTTATAGTTATACCTATTATAATTGGAGGATTTGGAAACTGACTTGTACCTTTAATACTTGGTGCACCCG
ATATAGCATTCCCACGAATAAATAATATAAGATTTTGAATACTACCACCTTCTTTAACTCTTTTAATTTCTAGAA
GTATAGTAGAAAATGGGGCAGGAACAGGGTGAACAGTTTATCCCCCACTTTCTTCTGGAATTGCACATGCAGG
AGCTTCTGTTGATTTAGCAATTTTTTCTTTACATTTAGCAGGAATTTCATCTATTTTAGGAGCTGTAAATTTTATT
ACTACTGTAATTAATATACGATCACCAGGAATTACTTTAGATCGAATACCTTTATTTGTATGATCAGTAGTAATT
ACAGCTATTTTATTATTATTGTCTTTGCCTGTTTTAGCTGGAGCAATTACTATATTATTAACAGATCGAAATCTA
AATACTTCATTTTTCGATCCAGCCGGAGGAGGGGATCCAATTTTATACCAA

>COI-3

TACTTTATATTTTATTTTTGGAGCTTGAGCCGGAATAGTAGGAACTTCTTTAAGTATTCTAATTCGAGCTGAATT
AGGTCATCCTGGTGCTTTTATTGGAGATGATCAAATTTATAATGTTATTGTAACAGCACATGCTTTTATTATAAT
TTTCTTTATAGTAATACCTATTATAATTGGGGGATTTGGAAATTGATTAGTTCCTTTAATATTAGGAGCCCCTGA
TATAGCTTTCCCTCGAATAAATAATATAAGATTTTGAATATTACCTCCTTCATTAACTTTATTAATTTCTAGAAGT
ATAGTAGAAAATGGAGCAGGGACAGGATGAACTGTATATCCTCCTCTTTCATCTGGAATTGCTCATGCTGGAG
CATCAGTTGATTTAGCTATTTTTTCATTACATTTAGCAGGTATTTCTTCAATTTTAGGAGCAGTAAATTTTATTAC
AACTGTTATTAATATACGATCTCCTGGAATTACATTAGATCGAATACCTTTATTTGTTTGATCCGTAGTAATTAC
AGCAGTATTATTATTATTATCTTTACCTGTATTAGCTGGAGCTATTACTATATTATTAACAGATCGAAATTTAAA
TACTTCTTTCTTTGACCCAGCCGGAGGAGGAGATCCAATTTTATACCAA

>COI-4

AACTTTATACTTTATTTTCGGTGCTTGAGCTGGAATAGTAGGAACATCTTTAAGAATCTTAATTCGAGCCGAAC
TAGGACATCCAGGAGCATTTATTGGAGATGATCAAATTTATAATGTAATTGTAACTGCTCATGCTTTTATTATA
ATTTTCTTTATGGTAATACCAATCATAATTGGAGGATTTGGAAATTGATTAGTTCCTTTAATATTAGGAGCCCCT
GATATAGCTTTTCCTCGAATAAATAACATAAGATTTTGAATACTTCCTCCTTCATTAACTCTTTTAATTTCTAGTA
GTATAGTAGAAAATGGGGCAGGAACAGGTTGAACTGTTTATCCTCCTCTATCATCAGGAATTGCTCATGCCGG
GGCTTCTGTAGATTTAGCAATTTTTTCACTTCATCTAGCTGGAATTTCTTCAATTTTAGGGGCCGTAAATTTTATT
ACAACAGTTATTAATATACGATCTCCTGGAATTACTTTAGATCGAATACCATTATTTGTATGATCTGTTGTAATT
ACTGCAGTATTATTATTATTATCATTGCCTGTATTAGCAGGAGCTATTACTATATTATTAACAGATCGAAACTTA
AATACATCATTCTTTGACCCTGCGGGAGGAGGAGATCCAATTTTATACCAA

>COI-5

AACATTATACTTTATTTTCGGAGCTTGAGCCGGAATAGTAGGAACTTCTTTAAGAATTTTAATTCGAGCTGAAT
TAGGTCACCCAGGAGCTTTTATTGGAGATGATCAAATTTATAATGTAATTGTAACAGCACATGCTTTTATTATA
ATTTTTTTTATAGTTATACCAATTATAATTGGAGGATTTGGAAATTGATTAGTTCCTTTAATATTAGGAGCTCCA
GATATAGCTTTCCCACGAATAAATAATATAAGATTTTGAATACTTCCTCCTTCATTAACTCTTCTTATTTCTAGAA
GTATAGTAGAAAATGGGGCTGGTACAGGATGAACAGTATACCCTCCTTTATCTTCTGGAATTGCTCATGCTGG
AGCTTCAGTAGATTTAGCTATTTTTTCTTTACATTTAGCTGGAATTTCTTCAATTTTAGGAGCTGTAAATTTTATT
ACTACTGTTATTAATATACGATCTCCTGGAATTACTTTAGATCGAATACCTTTATTTGTATGATCAGTAGTAATT
ACTGCTATTTTATTATTATTATCTTTACCTGTTTTAGCTGGAGCTATTACAATATTATTAACAGATCGAAATTTAA
ATACTTCATTTTTTGATCCTGCTGGAGGAGGAGATCCTATTTTATATCAA

>COI-6

AACATTATACTTTATTTTCGGTGCTTGAGCTGGAATAGTAGGAACTTCACTAAGAATTCTTATTCGAGCTGAAT
TAGGTCATCCCGGAGCATTTATTGGAGATGATCAAATTTATAATGTAATTGTTACTGCTCATGCTTTTATTATAA
TTTTCTTTATAGTTATACCTATTATAATTGGAGGATTTGGAAACTGACTTGTACCTTTAATACTTGGTGCACCCG
ATATAGCATTCCCACGAATAAATAATATAAGATTTTGAATACTACCACCTTCTTTAACTCTTTTAATTTCTAGAA
GTATAGTAGAAAATGGAGCAGGAACAGGGTGAACAGTTTATCCCCCACTTTCTTCTGGAATTGCACATGCAGG
AGCTTCTGTTGATTTAGCAATTTTTTCTTTACATTTAGCAGGGATTTCATCTATTTTAGGAGCTGTAAATTTTATT
ACTACTGTAATTAATATACGATCACCAGGAATTACTTTAGATCGAATACCTTTATTTGTATGATCAGTAGTAATT
ACAGCTATTTTATTATTATTGTCTTTGCCTGTTTTAGCTGGAGCAATTACTATATTATTAACAGATCGAAATCTA
AATACTTCATTTTTCGATCCAGCCGGAGGAGGGGATCCAATTTTATACCAA

References

1 Orba, Y. *et al.* First isolation of West Nile virus in Zambia from mosquitoes. *Transbound Emerg Dis* **65**, 933-938, doi:10.1111/tbed.12888 (2018).

2 Wastika, C. E. *et al.* Discoveries of exoribonuclease-resistant structures of insect-specific flaviviruses isolated in Zambia. *Viruses* **12**, doi:10.3390/v12091017 (2020).
