# Supplementary material for: Temporally consistent predominance and distribution of secondary malaria vectors in the Anopheles community of the upper Zambezi floodplain
Source: Sci Rep. 2022 Jan 7;12:240. doi: 10.1038/s41598-021-04314-4 (PMC8742069; doi:10.1038/s41598-021-04314-4)
Supplement: Supplementary file 2 — Supplementary Information 2. [file 41598_2021_4314_MOESM2_ESM.pdf]

Cross, D.E., Healey, A.J.E., McKeown, N.J., Thomas, C.J., Macarie, N.A., Siaziyu, V., Singini, D., Liywalii, F., Sakala, J., Silumesii, A. & Shaw, P.W.

# Supplementary Information: Adult mosquito data derived from previous studies

## Methods

Mosquito collections were undertaken in Mongu District, Western Province, Zambia, between 2012-2017 as reported in Orba *et al.* (2018)[1] and Wastika *et al.* (2020)[2]. Additional trapping following the same sampling protocol was undertaken in 2018 and 2019 (Orba *et al.,* 2021, pers. comm.).

Three CDC light traps (John W. Hock Co., FL, USA) baited with $CO_2$ produced by yeast fermentation, and three BG-Sentinel traps (Biogents, Germany) with BG-Lure synthetic attractant were deployed at two locations: at the floodplain edge immediately west of Mongu, and immediately west of Namushakende. Trapping was undertaken mainly outdoors, from afternoon until 10:00 the following morning for five nights on average in each sampling round. Mosquitoes were identified morphologically following identification keys of African mosquitoes[3,4], and up to 40 mosquitoes per species pool stored at -80°C.

A sample from each morphologically-identified pool was amplified by PCR and the cytochrome oxidase I gene sequenced[5] as a standard DNA barcoding for molecular identification. Species identities were confirmed by matching against NCBI GenBank and BOLD genetic databases. COI sequences of 6 species pools are included as Supplementary Information: Dataset 1.

## Results

**Table S1: Composition of adult anopheline mosquitoes sampled in Mongu, Zambia 2014-2019**

| | *An. gambiae s.l.* | *An. coustani* | *An. squamosus* | *An. species 2* | *An. species* | **TOTAL** |
|---|---|---|---|---|---|---|
| October 2014 | 0 | 19 | 0 | 0 | 0 | 19 |
| % | *0* | *100* | *0* | *0* | *0* | *100* |
| May 2016 | 0 | 182 | 54 | 124 | 6 | 366 |
| % | *0* | *49.7* | *14.8* | *33.9* | *1.6* | *100* |
| May 2017 | 7 | 709 | 173 | 0 | 555 | 1444 |
| % | *0.5* | *49.1* | *12.0* | *0* | *38.4* | *100* |
| August 2018 | 0 | 2 | 0 | 0 | 0 | 2 |
| % | *0* | *100* | *0* | *0* | *0* | *100* |
| December 2018 | 0 | 33 | 19 | 0 | 4 | 56 |
| % | *0* | *58.9* | *33.9* | *0* | *7.1* | *100* |
| May 2019 | 0 | 300 | 73 | 0 | 165 | 538 |
| % | *0* | *55.8* | *13.6* | *0* | *30.7* | *100* |
| **Species total** | **7** | **1245** | **319** | **124** | **730** | **2425** |
| % | *0.3* | *51.3* | *13.2* | *5.1* | *30.1* | *100* |

Trapping undertaken by Orba *et al.* (2018)[1] and Wastika *et al.*, (2020)[2]. Species identified morphologically, with molecular confirmation by sequencing of COI mtDNA for reference against published accessions on GenBank.

## References

1 Orba, Y. *et al.* First isolation of West Nile virus in Zambia from mosquitoes. *Transbound Emerg Dis* **65**, 933-938, doi:10.1111/tbed.12888 (2018).
2 Wastika, C. E. *et al.* Discoveries of exoribonuclease-resistant structures of insect-specific flaviviruses isolated in Zambia. *Viruses* **12**, doi:10.3390/v12091017 (2020).
3 Gillett, J. D. *Common African mosquitoes and their medical importance*. (William Heinemann Medical Books Ltd., 1972).
4 Kent, R. J. *The Mosquitoes of Macha, Zambia*. (John Hopkins Malaria Research Institute, 2006).
5 Folmer, O., Black, M., Hoeh, W., Lutz, R. & Vrijenhoek, R. DNA primers for amplification of mitochondrial cytochrome c oxidase subunit I from diverse metazoan invertebrates. *Molecular Marine Biology and Biotechnology* **3**, 294-299 (1994).
