# Supplementary material for: Temporally consistent predominance and distribution of secondary malaria vectors in the Anopheles community of the upper Zambezi floodplain
Source: Sci Rep. 2022 Jan 7;12:240. doi: 10.1038/s41598-021-04314-4 (PMC8742069; doi:10.1038/s41598-021-04314-4)
Supplement: Supplementary file 3 — Supplementary Information 3. [file 41598_2021_4314_MOESM3_ESM.pdf]

Temporally consistent predominance and distribution of secondary malaria vectors in the *Anopheles* community of the upper Zambezi floodplain

Cross, Dónall Eoin, Healey, Amy J.E., McKeown, Niall J., Thomas, Christopher James, Macarie, Nicolae Adrian, Siaziyu, Vincent, Singini, Douglas, Liywalii, Francis, Sakala, Jacob, Silumesii, Andrew & Shaw, Paul W.

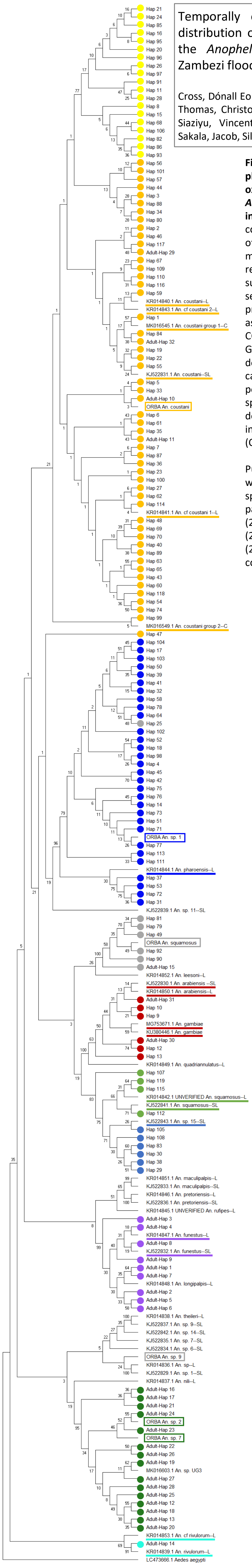

Figure S1: Maximum Likelihood phylogenetic tree of cytochrome *c* oxidase I (COI) sequences from *Anopheles* larvae and adults sampled in western Zambia. Consensus tree constructed in MEGA X from haplotypes of a 300bp fragment of the COI region of mitochondrial DNA, using 100 bootstrap replicates. Larval sequences from 2019 surveys have prefix 'Hap', and adult sequences from 2019 trapping have prefix 'Adult-Hap'. Species identities assigned from >95% BLAST similarity to COI and ITS2 sequences published on GenBank are indicated with coloured dots. Sequences from adult mosquitoes caught in the region (Orba *et al.*, 2021, pers. comm.) are prefixed 'ORBA'; species name indicates morphologically-derived identity, while coloured box indicates molecularly-derived identity (COI sequence).

Published reference sequences labelled with GenBank accession number and species name; suffix denotes source paper. '--C' denotes Ciubotariu *et al.* (2020)[37]; '--L' denotes Lobo *et al.* (2015)[19]; '--SL' denotes St Laurent *et al.* (2016)[20]. Species also indicated with coloured underline.
